# Closure of oroantral communications with Bichat´s buccal fat pad. Level of patient satisfaction

**DOI:** 10.4317/jced.51730

**Published:** 2015-02-01

**Authors:** Rocío Alonso-González, María Peñarrocha-Diago, David Peñarrocha-Oltra, Amparo Aloy-Prósper, Fabio Camacho-Alonso, Miguel Peñarrocha-Diago

**Affiliations:** 1 Master in Oral Surgery and Implant Dentistry, Stomatology Department. Faculty of Medicine and Dentistry. University of Valencia, Spain; 2 Full Professor of Oral Surgery. Stomatology Department. Faculty of Medicine and Dentistry. University of Valencia, Spain; 3 Master in Oral Surgery and Implant Dentistry. Collaborating Professor of Oral Surgery, Stomatology Department. Faculty of Medicine and Dentistry. University of Valencia, Spain; 4 Full Professor of Oral Surgery. Stomatology Department. Faculty of Medicine and Dentistry. University of Murcia, Spain; 5 Professor and Chairman of Oral Surgery and Implantology, Valencia. University Medical and Dental School, Valencia, Spain

## Abstract

Purpose: To report the closure of oroantral communications with the pedicled buccal fat pad in a series of patients, and to determine the level of patient satisfaction after the surgery. Study Design: A prospective study of patients diagnosed of unilateral or bilateral oroantral communication (OAC) closed using the buccal fat pad between May 2012 and January 2013 was performed. Data analysis extended to: age, sex, and cause, location and size of oroantral communication. Complications and success related to buccal fat pad surgery were evaluated. Also, patient satisfaction was assessed after six months of surgery. Results: Nine patients (3 men and 6 women) with a mean age of 50.5 years and 11 OAC treated with buccal fat pads were included. The most common cause of oroantral communication was the extraction of molars. The average widest diameter of the oroantral communication was 7.1 mm. One week after the surgeries no complications were found. One month after surgery, one patient presented persistence of the oroantral communication; in this patient, the buccal fat pad technique was considered a failure, and a second intervention was performed using a buccal mucoperiosteal flap to achieve primary closure of soft tissues. After six months, patient showed closure of the communication and complete healing. All the other communications had been solved with Bichat´s ball technique, yielding a success rate of 90.9%. Mean patient overall satisfaction was 9.1 out of 10; patients were satisfied with phonetics (9.4), aesthetics (9) and chewing (9). Conclusions: The buccal fat pad technique was successful in closing 10 out of 11 oroantral communications and few complications were found. Patients were highly satisfied in overall with the treatment and with phonetics, aesthetics and chewing.

** Key words:**Bichat’s fat pad, buccal fat pad, oroantral communication.

## Introduction

An oroantral communication (OAC) is an open connection between the oral cavity and a maxillary sinus (1). Its appearance is relatively common in oral surgery, caused by either simple or surgical extraction of antral teeth, cysts and tumors, or infectious processes (2). Oroantral communications less than 2 mm in diameter tend to close spontaneously, whereas those larger than 3mm require surgical closure (3). Numerous techniques for their closure have been described, including proximity or distance grafts and flaps, such as the pedicled Bichat´s ball (3,4).

Since Egyedi (5) described in 1977 the technique of closure of oroantral communications using pedicled Bichat´s ball, it has become a useful procedure in regenerative oral surgery. In the past three decades, several authors have resorted to using the Bichat´s ball to close oroantral communications of diverse etiology (6-12) either acute, chronic or of recurring character (10). Reported advantages of its use are the easy availability of the flap and the large blood supply that the recipient bed receives, which result in high success rates (7,13,14). Complications with this technique are rare (2,3), resulting in most cases aesthetic, phonetic and chewing acceptable results according to the authors. However, no studies were found in which patient level satisfaction after the surgery were evaluated.

The objective was to report the closure of oroantral communications with the pedicled buccal fat pad in a series of nine patients, and to determine the level of patient satisfaction after the surgery.

## Material and Methods

The present study is reported in accordance with the STROBE statement for strengthening the reporting of observational studies (15).

-Study design and patient selection 

A prospective study of patients with unilateral or bilateral oroantral communications treated in the Oral Surgery Unit of the University of Valencia between May 2012 and January 2013 was performed. Inclusion criteria were: age > 18 years, absence of relevant medical conditions, non-smoking or smoking ≤ 10 cigarettes/day and possibility for follow-up for 6 months after surgery. Patients with sinus pathology were excluded. A total of 11 patients with oroantral communications were consecutively included in the study. The study was approved by the local ethics committee (Ref. H13355958803910), and followed the principles of the Declaration of Helsinki for human research. All patients gave written informed consent before surgery.

-Surgical technique 

**Figure 1:**
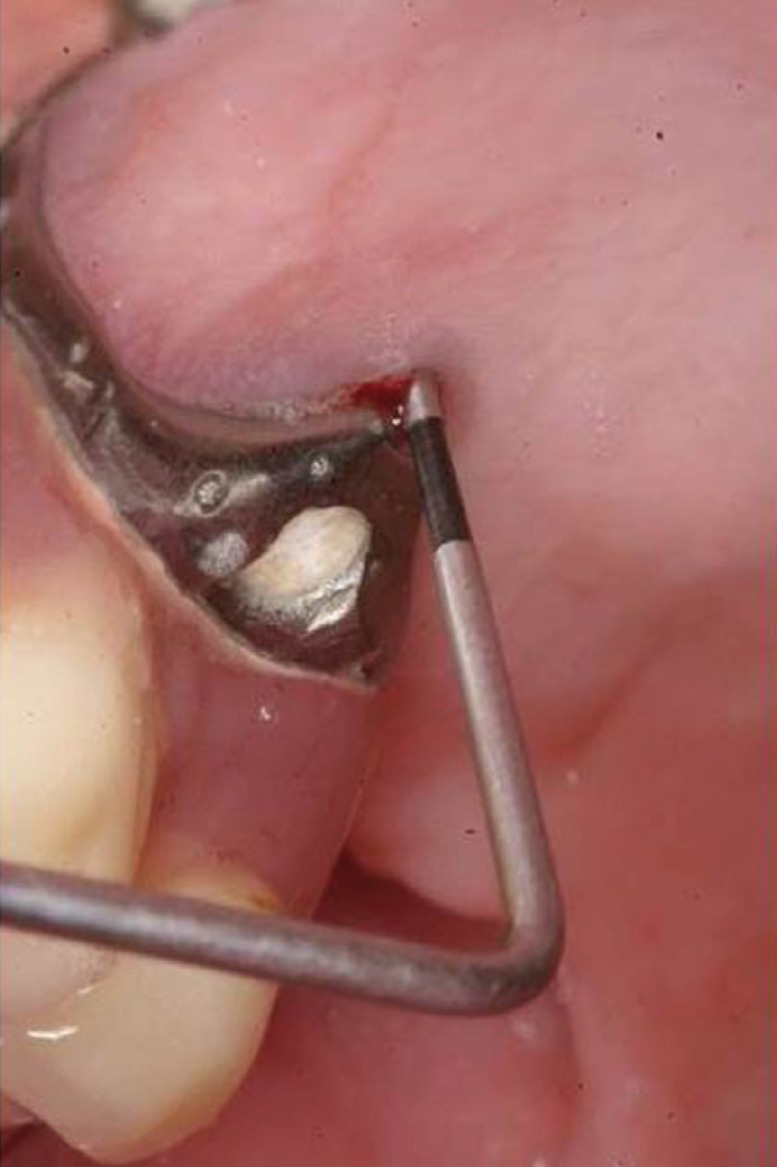
Detail of probing depth of the failed transzygomatic implant showing oroantral communication.

**Figure 2:**
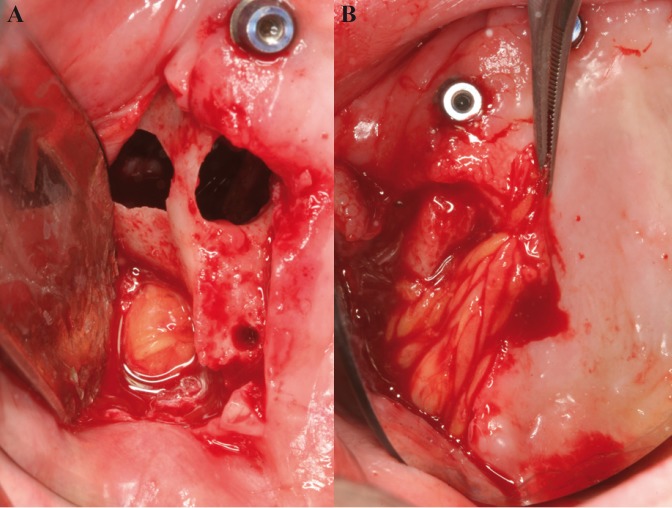
A) After failed implant removal, full-thickness mucoperiosteal flap is elevated; the bone defect and oroantral communication can be observed. B) Pedicled fat pad is located over the defect.

**Figure 3:**
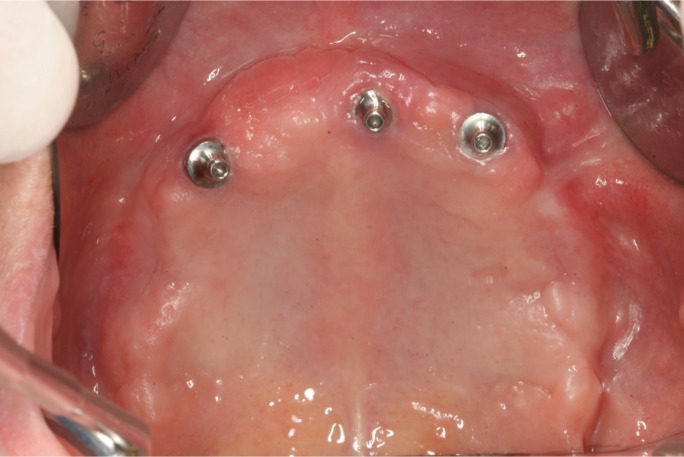
Soft tissue healing 6 months after surgery; closure of the communications is achieved.

All surgical procedures were performed under local anesthesia with articaine 4% and adrenaline 1:100.000 (Inibsa ®, Lliça Vall, Barcelona, Spain) by the same surgeon (MPD). After COA occurrence, buccal fat pad technique was selected in order to its seal. To expose the buccal fat pad, in all cases a trapezoidal mucoperiosteal flap was raised extending on each side of the defect and to the bottom of the vestibule. A 1-cm vertical incision was made in the reflected periosteum, posterior to the zygomatic buttress. A blunt clamp was introduced towards the temporomandibular angle to separate the fibers of the buccinator muscle, and a light pressure was exerted on the cheek to prolapse the buccal extension of Bichat´s ball. The necessary amount of buccal fat was pedicled to cover the defect entirely (Figs. 1-3). Once placed on the defect, the fat pad was covered as much as possible with the mucoperiosteal flap because due to its fragile and lobulated structure the buccal fat pad alone may not always provide adequate sealing. The mucoperiosteal flap was sutured without tension.

-Postoperative care 

Amoxicillin 500 mg and Ibuprofen 600 mg were prescribed to be taken three times daily for 7 days. Patients were also instructed to rinse with 0.12% chlorhexidine digluconate three times daily for two weeks following surgery. A soft diet was recommended for one week and patients were instructed to avoid brushing or any other trauma to the surgical sites. Sutures were removed one week after surgery.

-Data collection and follow-up

Patient age and gender were collected. Regarding the oroantral communication, the following data were assessed: cause (simple or surgical dental extraction, cystectomy or extraction of failed implant), location (first or second quadrant; premolar, molar zone or both), size (largest diameter in millimeters) and intraoperative complications (bleeding and/or pain). The communication size was determined by measuring the mesio-distal and bucco-palatal dimensions of the bony defect through a periodontal probe after raising the mucoperiosteal flap; the largest diameter was considered.

•Receptor site healing: Control visits were performed one week, one month and six months after the surgeries. Postoperative complications were collected one week (hematoma, wound dehiscence, oroantral communication persistence, and local infection), one and six months after the surgery (oroantral communication persistence, phonetic/chewing limitations, and facial cosmetic defect).

•Surgical technique success: The technique was considered successful if closure of the oroantral communication was achieved and no recurrence occurred, tested by a negative “nose blowing” in the successive control visits.

•Patient satisfaction: At the six-month follow-up, overall patient satisfaction regarding the treatment and specific satisfaction with phonetics, aesthetics and chewing were assessed using 10-cm visual analogue scales (VAS). “Completely dissatisfied” and “completely satisfied” were used as anchor words at the ends of the VAS. Patients were asked to draw a vertical line at the point on the horizontal line that best represented their satisfaction. The distance from the left end of the lines to the drawn vertical line was measured with a millimitered ruler and recorded as level of satisfaction out of 10.

Statistical analysis was done using SPSS 15.0 software for Windows (SPSS Inc., Chicago, IL). We performed a descriptive analysis of the variables studied, obtaining the means of central tendency and standard deviations.

## Results

Two out of initial 11 patients were excluded due to lack of 6-months follow-up. A total of nine patients (3 men and 6 women) with a mean age of 50.5 years (range 29 to 64) and eleven oroantral communications (2 bilateral cases) treated with buccal fat pads were included. The causes of communication were: 4 simple dental extractions (bilateral extractions in one patient), 4 surgical extractions, one cyst removal, and 2 failed transzygomatic implants in one patient ( Table 1; Figs. 1-3). The mean communication size was 7.1 mm in diameter (range 5 mm to 14mm). The largest communication (14mm) was caused by a failed implant removed from the right zygomatic buttress ( Table 1).

**Table 1:**
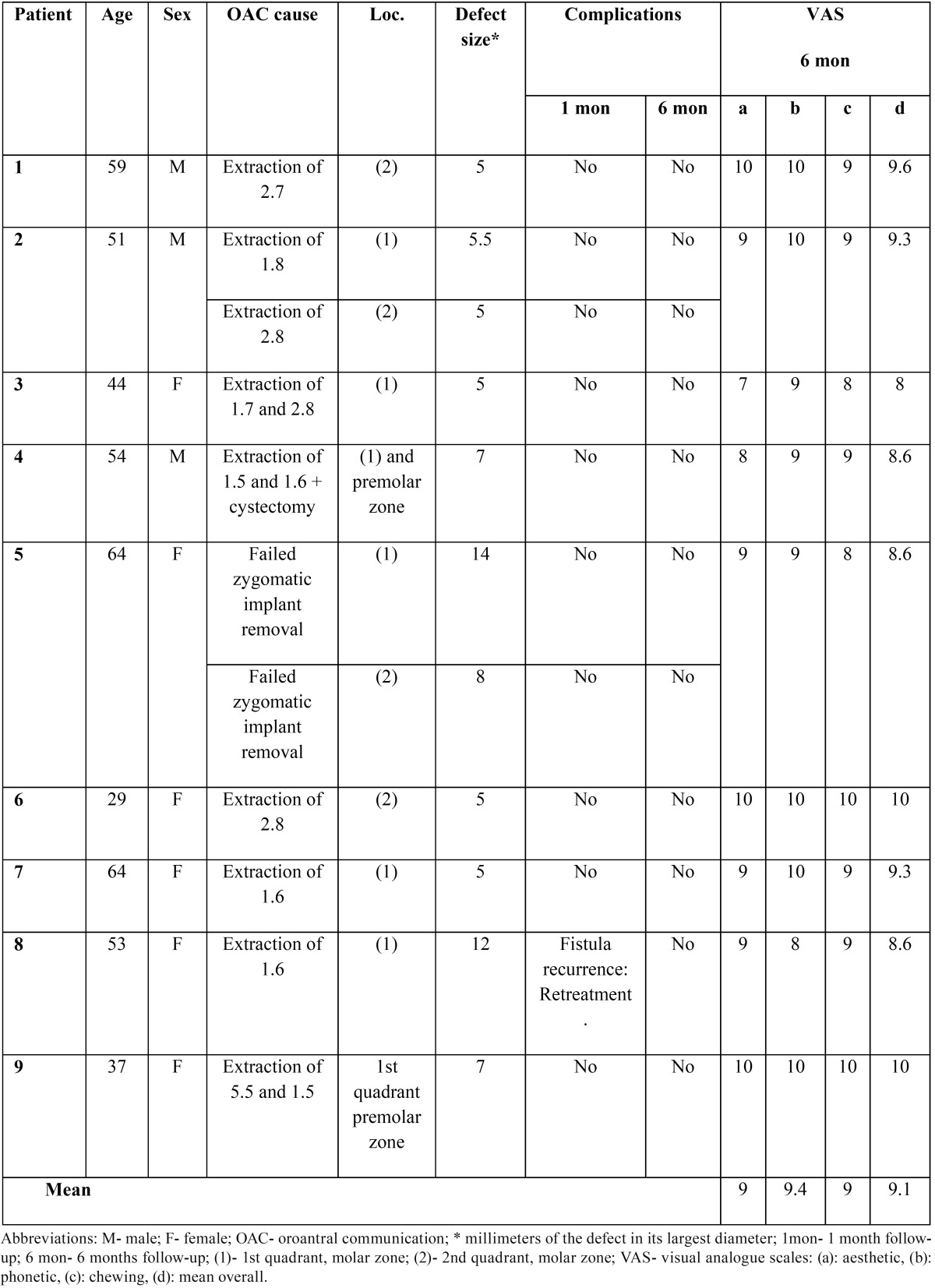
Study variables. Orosinusal communications.

In one patient an oroantral communication persisted one month after surgery. A second intervention was then performed, using buccal mucoperiosteal flap to achieve primary closure of the soft tissues. Six months after surgery, complete healing and closure of the communication was observed. No more postoperative complications were collected.

In summary, at the end of follow-up all oroantral communications except one (patient 8) were resolved with the Bichat´s pad technique, yielding a success rate of 90.9%. The mean overall satisfaction of patients was 9.1 out of 10 (range 8 to 10); satisfaction with the other assessed parameters are reported in  Table 1.

## Discussion

The use of the pedicled Bichat´s ball in the treatment of OAC and maxillary bone defects has been reported in several studies with good results (2,3,6-11).

The buccal fat pad has its own mechanism of lipolysis, unlike subcutaneous adipose tissue (7), so neither age nor sex of the patient are important in determining the outcome with this technique (4,7). For this reason good results have been reported with the buccal fat pad technique even in old patients (15).

Regardless of location, oroantral communications described in the literature were secondary to: tooth extraction (3,4,6,7,10,11), cystic and tumor excisions (6,7), sinus lifts (7,14), and periimplantitis (7). In this study, nine patients with eleven OAC (2 bilateral cases) treated with buccal fat pads were included, following the STROBE statement (16). The OAC causes were: 4 simple dental extractions and 4 surgical extractions, 1 cyst removal, and removal of 2 failed implants.

Oroantral communication treated with the Bichat’s ball in the literature range from 2 mm (8) to 50 mm in diameter (14,16). Abuabara *et al.* (3) recommended the use of the Bichat´s ball in large communications (> 5 mm in diameter), in which the blood supply of a buccal flap could be compromised and/or loss of vestibular sulcus depth could occur. However, the most critical factor for the success of the buccal fat pad seems to be the size of the oroantral communication (13). The pedicled Bichat´s ball technique has also limitations in large defects because their closure requires traction of a greater portion of the ball, which increases the likelihood of postoperative complications such as aesthetic depression of the cheek (7). Alkan *et al.* (14) reported successful closure of bony defects up to 50 x 30 mm in area; similarly Rapidis *et al.* (16) recommended limiting the use of the pedicled Bichat´s ball to defects under 40 x 40 mm. There is lack of consensus within the literature on how the communications are measured: maximum diameters have been reported in millimeters (3,10,11), bone defect areas in millimeters (2,14), and even bone defect volumes in millimeters (3,15,17). In the present study, the largest diameter of the oroantral communication was measured with a periodontal probe and taken as reference. Sizes ranged from 5 mm to 14 mm in diameter.

The literature collect intra and postoperative complications related to oroantral communication closure using the buccal fat pad (4,6,12,15,17-19). Although infection is mentioned as a potential complication, analysis of the reported cases proved that only three cases have been described (0.82%). The most common complications in the literature was persistence of the communication and mouth opening limitation, especially in cases of oroantral communications accompanied by large bone defects (6-8). In this study, only one patient had communication persistence, which was diagnosed one month after surgery.

Success of the buccal fat pad technique has been attributed to its rich vascular supply, less donor site morbidity, almost constant weight for all individuals, reliability, ease of harvest and low complication rate (15). Alkan *et al.* (14) defined their success criterion: complete epithelialisation of the graft, and neither infection of the graft, fistulae recurrence nor facial contour deficiency. Although not all authors defined success criteria, it is assumed that the buccal fat pad technique was successful when the oroantral communication was closed at the end of the time of follow-up, which in the literature ranged from 4 weeks to 62 months (7,13). However, according to Poeschl *et al.* (7), from 3 to 6 months of follow-up is sufficient to assess the success of healing. In this study, the technique was considered successful if closure of the communication was achieved and no recurrence occurred (tested by a negative “nose blowing” in the successive controls one week, one month and six months after the surgery). One recurrence was observed one month after the surgery and this case was considered a failure; therefore, after six months follow-up, the success rate was 90.9%. Due to aesthetic and functional complications described in the literature derived from bucal fat pad´s handling, authors considered interesting to evaluate the patient satisfaction degree after treatment. At the six-month follow-up, overall patient satisfaction regarding the treatment and specific satisfaction with phonetics, aesthetics and chewing were assessed using 10-cm visual analogue scales (VAS). “Completely dissatisfied” and “completely satisfied” were used as anchor words at the ends of the VAS (20-22). To our knowledge, the present study is the first series of oroantral communications treated with the Bichat´s ball which reports the patient level satisfaction after the surgery, overall and regarding aesthetics, phonetics and chewing. The mean average overall satisfaction 6 months after the surgery was 9.1 out of 10 (range 8 to 10), which was considered rewarding. Because of the high success rate and patient rate satisfaction, it was considered that the use of buccal fat pad is a suitable alternative to to the traditional buccal flap.

## Conclusions

The buccal fat pad technique was successful in closing 10 out of 11 oroantral communications and only one communication persistence was found as complication. Patients were highly satisfied in overall with the treatment and with phonetics, aesthetics and chewing.
